# Development of Drug Efficacy Testing Platform for Glomerulonephritis

**DOI:** 10.3390/mi15030317

**Published:** 2024-02-24

**Authors:** Eun-Jeong Kwon, Yunyeong Choi, Shin Young Kim, Seokwoo Park, Giae Yun, Sei Hong Min, Sejoong Kim

**Affiliations:** 1 Department of Internal Medicine, Seoul National University Bundang Hospital, Seongnam-si 13620, Republic of Korea; dolce102003@gmail.com (E.-J.K.); dbsdud200@hanmail.net (Y.C.); chlorella@hanmail.net (S.Y.K.); no1seokwoo@gmail.com (S.P.); giaeyun0106@gmail.com (G.Y.); titotio@naver.com (S.H.M.); 2 Department of Internal Medicine, Seoul National University College of Medicine, Seoul 03080, Republic of Korea

**Keywords:** 3D glomeruli tissue chip, glomerular filtration barrier mimicking chip, drug efficacy platform, MN chip

## Abstract

We developed a 3D glomeruli tissue chip for glomerulonephritis (GN) testing, featuring a gravity-driven glomerular filtration barrier (GFB) with human podocytes and endothelial cells with a bidirectional flow in the bottom channel. Using puromycin-induced GN, we observed decreased cell viability, increased albumin permeability, and reduced WT1 and nephrin compared to the normal GFB. Tacrolimus restored cell viability, reduced albumin permeability, and increased WT1 expression. Using serum from five membranous nephropathy (MN) patients, we created MN models using a GFB-mimicking chip. A notable decline in cell viability was observed in the serum-induced MN1 and MN2 models. However, tacrolimus restored it. Albumin permeability was reduced in the MN1, MN2, and MN5 models by tacrolimus treatment. MN1 displayed the best clinical response to tacrolimus, exhibiting increased expression of WT1 in chip-based evaluations after tacrolimus treatment. We successfully evaluated the efficacy of tacrolimus using puromycin-induced and serum-induced GN models on a chip that mimicked the structure and function of the GFB. The GFB-mimicking chip holds promise as a personalized platform for assessing drug efficacy using patient serum samples.

## 1. Introduction

MN is a glomerular disease that accounts for 30% of adult nephrotic syndrome cases [1]. It is characterized by the presence of auto-circulating antibodies that recognize protein on podocytes, leading to the destruction of the glomerular filtration barrier (GFB). The decision to initiate treatment for glomerular disease is determined by patient risk and disease progression, aligning with Kidney Disease Improving Global Outcomes (KDIGO) guidelines, which recommend the selection of immunosuppressive agents. Rituximab is proposed as the first therapeutic option for MN [2]. However, due to cost and public insurance considerations, alternatives such as calcineurin inhibitors, tacrolimus and cyclosporin, cyclophosphamide, and steroids may be chosen. Patients with MN show significant variability in how they respond to medications, underscoring the need for precise, personalized treatment strategies tailored to each patient’s unique characteristics. However, as of now, no platform capable of evaluating which medication is most suitable for a patient exists. Moreover, even with the use of specific medications, achieving complete remission of proteinuria typically requires about 6 months from the start of treatment, alongside careful consideration of the potential side effects associated with each medication. Consequently, there is a practical need in clinical settings for a system that can experimentally predict a personalized drug for the patient and advance screening methods for effective therapies to enhance the prognosis of individuals with glomerulopathy.

Traditional in vitro and in vivo animal experiments are common models for studying the therapeutic effects of drugs in various diseases. Animal testing is employed in pharmaceutical and industrial research to predict human toxicity. However, due to the inability of animal models to accurately mimic human physiology in predicting drug safety, their reliability is compromised, leading to significant drawbacks, such as prolonged experimental periods and delays in drug approval [3]. In particular, two-dimensional (2D) experimental models fail to replicate continuous exposure to shear stress in the glomerulus. Due to this limitation, we aimed to create a GFB-mimicking chip by applying physiological shear stress using the 3D-multi-organ tissue interdisciplinary value escalation (3D-MOTIVE) chip introduced in our previous study [4]. As reported, the incorporation of physiological fluidic shear stress allows us to address the shortcomings of traditional models [5,6]. We fabricated a GFB-mimicking chip and through this chip, we replicated the MN model to assess the efficacy of tacrolimus in treating MN, with a specific focus on its impact on glomerular damage associated with podocytopathies.

## 2. Materials and Methods

### 2.1. Cell Culture

Human podocytes (CIHP-1, Ximbio, UK, CVCL_W186) were cultured in RPMI-1640 medium (Gibco, Grand Island, NY, USA, #11875093) with 10% fetal bovine serum (FBS, Gibco, Grand Island, NY, USA, #16000-044), 1% insulin–transferrin–selenium (ITS, Gibco, Grand Island, NY, USA, #41400-045), and 1% penicillin–streptomycin (Gibco, Grand Island, NY, USA, #15140-122) at 33 °C in a 5% CO_2_ incubator. Cells within passages 5–15 were used for the experiments. For podocyte differentiation, CIHP-1 cells were initially cultured at 33 °C to promote proliferation and then transferred to 37 °C for 7–14 days to induce differentiation. Primary human glomerular endothelial cells (RFP-HGMVECs, ANGIO-PROTEMIE, Boston, MA, USA, #cAP-0004RFP) were cultured in endothelial basal medium (EBM) (ANGIO-PROTEMIE, Boston, MA, USA, #cAP-03) consisting of 10% endothelial growth supplements (ANGIO-PROTEMIE, Boston, MA, USA, #cAP-04) and 1% penicillin–streptomycin.

### 2.2. GFB-Mimicking Chip in 3D-MOTIVE Chip and Glomerulonephritis Model

**Figure 1:**
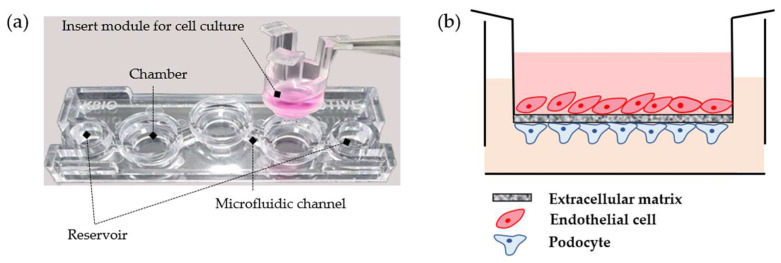
Schematic illustration of the experimental tools. (**a**) Design of the 3D-MOTIVE chip. (**b**) Design of the GFB-mimicking chip on 3D-MOTIVE.

The structure of the 3D-MOTIVE chip was previously introduced [4]. In brief, the 3D-MOTIVE chip (K-bio, Osong, Republic of Korea) features a single layer of SABIC Lexan^®^ 121R polycarbonate with three badge chambers and separate-fit inserts (Figure 1). Polycarbonate was chosen as the material for chip fabrication due to its affordability, superior impact resistance, minimal moisture absorption, and outstanding manufacturability [7]. The chip includes a main microfluidic component and a separatable insert module for cell culture. The inserts, equipped with a 0.4 µm pore size polyethylene–terephthalate membranes (Greiner BIO-ONE, Lagoas Park, Portugal #662641), were coated with collagen type I (Corning, Tewksbury, MA, USA #354265) before seeding with CIHP-1 cells at 1 *×* 10^5^ cells/mL. After 2 h, the CIHP-1 layer formed, and the insert was recoated with a quick coating solution (ANGIO-PROTEOMIE, Boston, MA, USA, #cAP-01) before seeding with RFP-HGMVECs at 2 × 10^5^ cells/mL. The medium inside the insert was then replaced with a 1:1 mixture of CIHP-1 and RFP-HGMVEC media. The glomeruli tissue chip produced through this process was continuously perfused on an Organo Flow L rocker (Mimetas, Gaithersburg, MD, USA #MI-OFPR-L) to establish a bidirectional flow [5]. This flow was initiated by adjusting the rocker to an angle of 7° with an interval of 8 min, resulting in a mean flow rate of 2.02 μL/min and a mean shear of 1.3 × 10^−6^ N/cm^2^. The culture medium was changed every 2–3 days.

Serum-free RPMI-1640 was introduced into the chip’s channel, while a mixture of serum-free RPMI-1640 and EBM at a 1:1 ratio was added to the insert. For the induction of the GN model, treatment with puromycin aminonucleoside (Sigma, Burlington, MA, USA, P7130) at a concentration of 1 mg/mL or 0.5% MN patient serum was carried out for 24 h. Normal human serum (Millipore, Billerica, MA, USA, #S1-100ML) served as the control. Serum-free RPMI-1640 medium supplemented with tacrolimus (Prograf^®^, 10 µg/mL; Astellas Pharma Inc., Seoul, Republic of Korea) was added to the channel of the chip on the GN model and incubated for 6 h. After 6 h, the TAC-supplemented media was removed from the chip, and cell viability, albumin permeability, and immunostaining assays were performed as described below.

### 2.3. Cell Viability Assay

Cells were incubated with 2 μM calcein acetoxymethyl ester (Calcein-AM, Invitrogen, Burlington, ON, Canada, #C3099) at 37 °C for 30 min and then washed with phosphate-buffered saline (PBS). Fluorescence images were observed using a confocal microscopy system (ZEISS LSM 800 Confocal Laser Scanning Microscope, Carl Zeiss, Jena, Germany). Three-dimensional images were captured utilizing Z-stack modes.

CCK8 (Dojindo, Kumamoto, Japan, #CK-04) was added to the cells and incubated for 1 h at 37 °C. Absorbance was measured at 450 nm using a microplate reader (SpectraMax iD3, Molecular Devices, San Jose, CA, USA).

### 2.4. Albumin Permeability

The transport of albumin from the upper endothelial to the lower podocyte compartment was quantified to evaluate the diffusional permeability of the chip. Albumin–fluorescein–isothiocyanate (FITC; Sigma-Aldrich, Burlington, MA, USA, #A9771) at a concentration of 0.1 mg/mL was introduced into the insert and incubated for 1 h at 37 °C. Following the incubation, samples from both the inside of the insert (endothelial cells) and the bottom of the channel (podocytes) were collected and dispensed into a black 96-well plate. Absorbance was measured using a microplate reader (SpectraMax iD3, Molecular Devices, San Jose, CA, USA) at emission/excitation wavelengths of 453/488 nm.

### 2.5. Immunofluorescence Staining

The cells were fixed with 4% paraformaldehyde (Biosesang, Yongin, Republic of Korea) for 20 min at room temperature. Subsequently, the fixed cells underwent PBS washes (three times for 5 min) and were permeabilized using a 0.2% Triton X-100 solution (Sigma-Aldrich, Burlington, MA, USA #T8787) for 20 min at room temperature. Following this, the cells were washed with PBS and blocked with 3% bovine serum albumin (BSA; Bovogen, East Keilor, Australia, #BSAS 0.1) for 40 min at room temperature. Primary antibodies for nephrin (1:50, Bioss, Woburn, MA, USA, #bs-10233R-FITC) and Wilms’ tumor 1 (WT1) (1:100, Bioss, Woburn, MA, USA, #bs-6983R-A647) were diluted in 1% BSA overnight in the dark on a rocker at RT. Donkey anti-rabbit IgG Alexa fluor 488 (1:200, Abcam, Cambridge, UK, #ab150073) was diluted in 1% BSA and incubated for 2 h in the dark on a rocker at RT. Actin Green 488 (2 drops per 1 mL in 1ⅩPBS, Invitrogen, Carlsbad, CA, USA, #R37110) was added and incubated for 30 min in the dark on a rocker at RT. Cell nuclei were counterstained with 4′,6-diamidino-2-phenylindole (DAPI) (1:500, Invitrogen, Carlsbad, CA, USA, #D1306) in 1ⅩPBS for 30 min in the dark on a rocker at RT. Fluorescence images were observed using a confocal microscopy system. Three-dimensional images were captured utilizing Z-stack modes.

### 2.6. Statistical Analysis

The SPSS statistical software version 22 package (SPSS, Inc., Chicago, IL, USA) was used to perform all statistical analyses. Data are presented as means ± standard deviation (SD) and analyzed using the student t-test or one-way analysis of variance (ANOVA) if normality was satisfied according to the Shapiro–Wilk test. If normality was not satisfied, the data were analyzed using the Mann–Whitney U-test to compare 2 groups or the Kruskal–Wallis test to compare 3 or more independent groups. One-way ANOVA, followed by Dunnett’s multiple-comparison test, was applied for multiple comparisons. *p*-values of less than 0.05 were considered statistically significant.

## 3. Results

### 3.1. Mimicking the GFB in Microfluidic Environments

**Figure 2:**
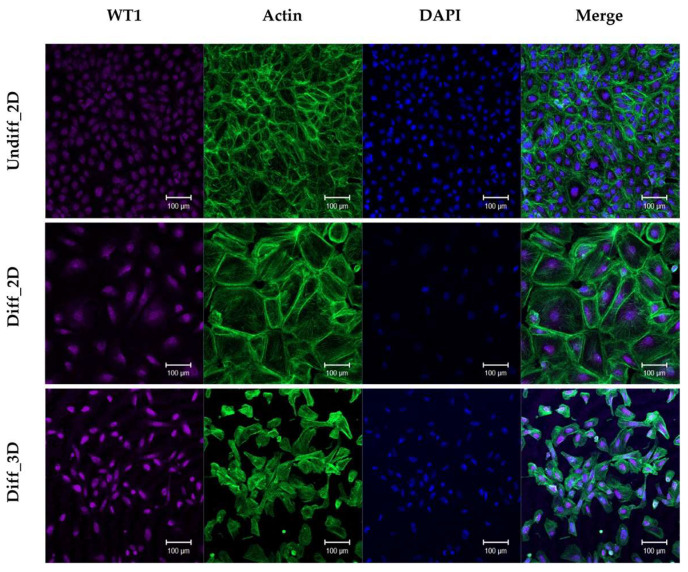
Morphology and WT1 expression on 2D plate and the GFB-mimicking chip. Abbreviations: Undiff_2D = undifferentiation with 2D culture; Diff_3D = differentiation with 3D GFB-mimicking chip (original magnification ×10, scale bar: 100 μm).

The manufacturing process for the GFB-mimicking chip is described in Section 2.2. Using the 3D-MOTIVE chip, we simultaneously cultured podocytes and endothelial cells on the membrane of the insert module (Figure 1). A rocker machine was employed to ensure continuous perfusion, allowing for the bidirectional flow of the medium [5]. A comparative analysis of the morphological changes in podocyte differentiation was conducted between culturing podocytes alone on a 2D plate and co-culturing them on the GFB-mimicking chip (Figure 2). The GFB-mimicking chip revealed a more well-differentiated form of podocytes, characterized by flat, arborized cells with well-developed prominent processes [8].

### 3.2. Tacrolimus Restores Cell Viability and Albumin Permeability in the Puromycin-Induced GN Model on the GFB-Mimicking Chip

**Figure 3:**
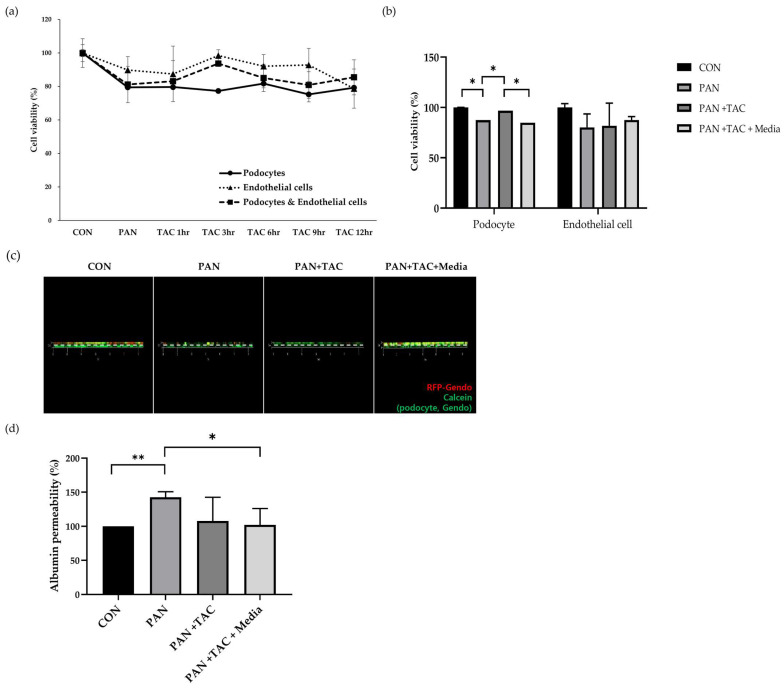
Cell viability and albumin permeability in the PAN-induced GN model on the GFB-mimicking chip. (**a**,**b**) CCK-8 assay. (**c**) Immunofluorescence staining of calcein-AM. (**d**) Albumin permeability (* *p* < 0.05, ** *p* < 0.01, data are presented as mean ± SD; n = 3). Abbreviations: PAN = puromycin aminonucleoside; TAC = tacrolimus.

Puromycin aminonucleoside (PAN) is a well-established drug known to induce podocyte injury both in vitro and in vivo [9,10]. This damage is frequently associated with F-actin disruption and glomerular dysfunction. Therefore, podocytes were treated with PAN for 24 h to create a chemical-induced GN model, followed by subsequent treatment with tacrolimus to assess its potential reversal effects. The viability of podocytes increased with the duration of tacrolimus treatment. However, the maximal reversal effect of dual cells was observed at 6 h, prompting the selection of a 6 h treatment duration for subsequent experiments (Figure 3a). Tacrolimus not only increased podocyte cell viability but also reduced albumin permeability by PAN-induced podocyte injury (Figure 3b–d).

### 3.3. Tacrolimus Increases Wilms’ Tumor-1 in the PAN-Induced GN Model on the GFB-Mimicking Chip

**Figure 4:**
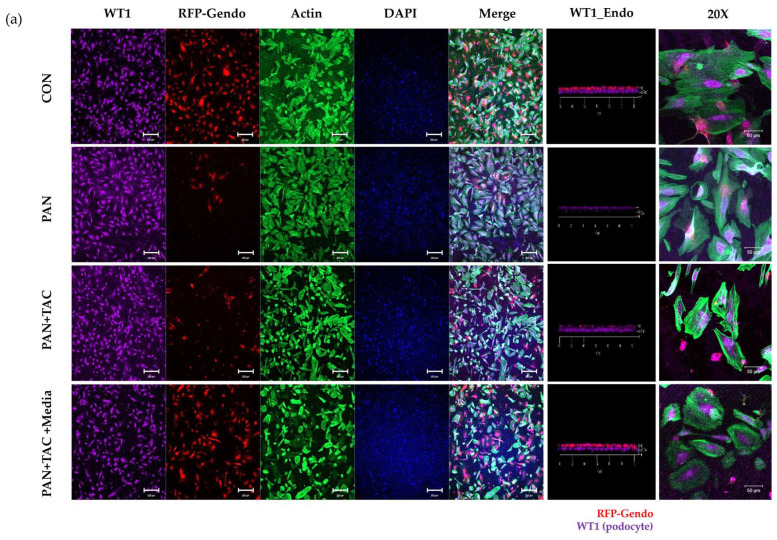

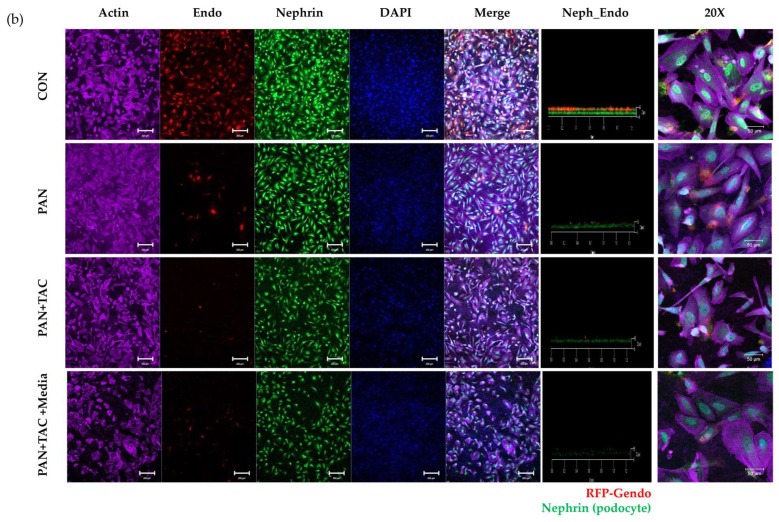
Immunofluorescence staining of WT1 and nephrin in the PAN-induced GN model on the GFB-mimicking chip. (**a**) WT1 expression. (**b**) Nephrin expression (original magnification ×4, scale bar: 200 μm and original magnification ×20, scale bar: 50 μm). Abbreviations: PAN = puromycin aminonucleoside; TAC = tacrolimus; Neph = nephrin; Endo = endothelial cell.

The expression of markers known to be crucial for these processes was examined using immunofluorescence staining in the GFB-mimicking chip to assess the maintenance of podocyte cytoskeleton and foot processes. PAN treatment decreased the expression of Wilms’ tumor-1 (WT1), a marker associated with podocyte maturation and glomerulogenesis [11]. Treatment with tacrolimus resulted in a subsequent increase in WT1 expression (Figure 4). The expression of nephrin, a marker associated with the maintenance of foot processes and slit diaphragm, decreased following PAN treatment. However, no restoration of nephrin expression was observed with tacrolimus treatment.

### 3.4. Clinical Characteristics and Pathological Features of Patients with MN

**Figure 5:**
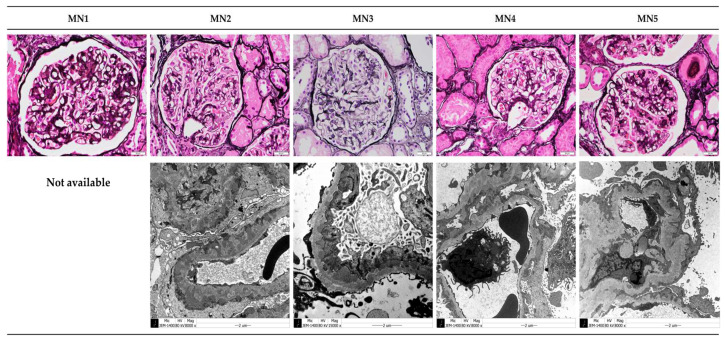
Pathological features of MN patients. The glomeruli in all patients with MN exhibited thickening of the GBM stained with periodic acid–methenamine silver (PAMS) by light microscopy (original magnification ×400, scale bar: 20 μm). In electron microscopy, EDDs were seen in the subepithelial space, and podocyte foot process effacement was seen in MN2, MN3, MN4, and MN5 patients (original magnification ×8000, ×15,000, scale bar: 2 μm).

We analyzed the clinical characteristics of patients registered for the creation of a serum-induced MN model using the serum of individuals with MN (Table 1). MN1 and MN5 were both positive for anti-PLA2R antibodies, and all patients exhibited massive proteinuria (UPCR ≥ 3500 mg/g). All kidney biopsies revealed diffuse capillary wall thickening, with IgG deposition of 2+ or higher observed in immunofluorescence microscopy. Electron microscopy showed diffuse podocyte foot process effacement and glomerular basement membrane (GBM) thickening (Figure 5). MN1, MN2, MN3, and MN5 patients were treated with tacrolimus. Complete remission was achieved with tacrolimus in MN1, while MN2 and MN5 showed partial remission. However, MN3 did not exhibit a therapeutic response to tacrolimus. MN4 had no history of treatment with tacrolimus.

### 3.5. Development of Drug Efficacy Platform in the Serum-Induced MN Model on the GFB-Mimicking Chip

**Figure 6:**
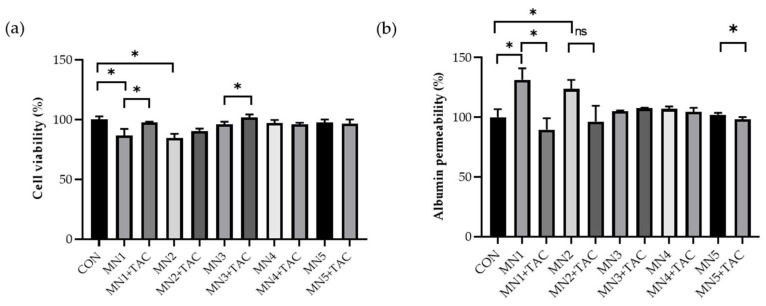
Cell viability and albumin permeability in MN serum-induced GN models on the GFB-mimicking chip. (**a**) CCK-8 assay. (**b**) Albumin permeability (* *p* < 0.05, data are presented as mean ± SD; n = 3). Abbreviations: ns = not significant.

Podocytes were treated with patient serum for 24 h to create a serum-induced MN model, followed by subsequent treatment with tacrolimus. The viability of podocytes increased with tacrolimus treatment in the MN1 and MN3 models. In the MN2 model, cell viability also increased with tacrolimus treatment, but the difference was not statistically significant (Figure 6a). Tacrolimus reduced albumin permeability in podocytes injured by MN1 and MN5 serum (Figure 6b). In the MN2 model, albumin permeability also decreased with tacrolimus treatment, but the change was not statistically significant.

**Figure 7:**
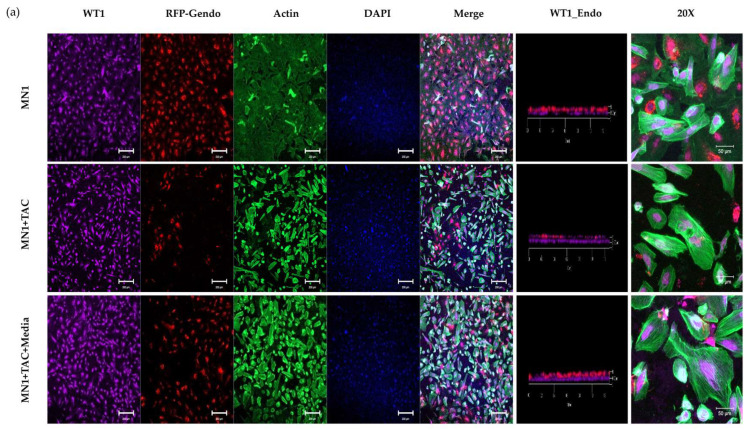

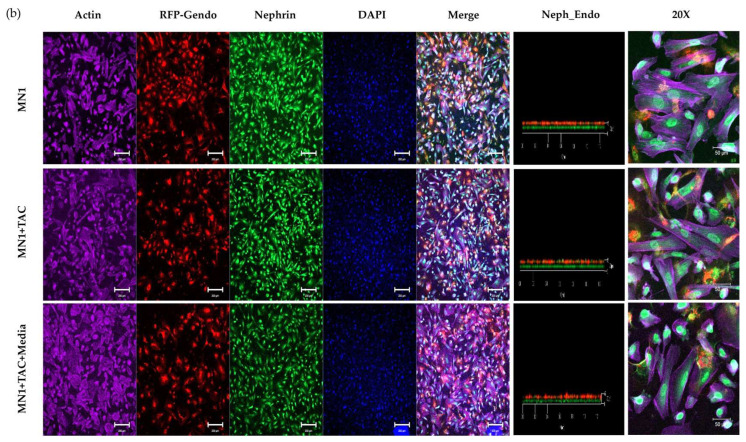
Immunofluorescence staining of WT1 and nephrin in the MN1 serum-induced GN model on the 3D glomeruli tissue chip. (**a**) WT1 expression. (**b**) Nephrin expression (original magnification ×4, scale bar: 200 μm and original magnification ×20, scale bar: 50 μm).

In the 3D glomerular tissue chip model, MN1 serum decreased WT1 and nephrin expression in podocytes (Figure 7). Upon treatment with tacrolimus, there was a subsequent increase in WT1 expression in the MN1 model. However, nephrin expression was not recovered in the MN1 model by tacrolimus treatment.

## 4. Discussion

We created a PAN-induced GN model and serum-induced MN model on a GFB-mimicking chip. In the PAN-induced GN model, podocytes exhibited reduced levels of WT1 and nephrin, resulting in increased albumin permeability and decreased cell viability compared to the standard GFB. After the administration of tacrolimus, WT1 expression increased in the PAN-induced GN model. Tacrolimus also led to a reduction in albumin permeability and the restoration of cell viability in this model. MN patients (MN1, MN2, and MN5) who received tacrolimus showed significant improvements clinically. In the MN models induced by MN1 and MN3 sera on the chip, there was a significant decrease in cell viability compared to other groups, which was restored after tacrolimus treatment. Albumin permeability decreased in the MN1 and MN5 models on the chip following tacrolimus treatment.

The glomerulus chip model can be divided into the following mechanisms [12]. The first is a chip with a porous membrane, made from a polydimethylsiloxane plate with the porous membrane serving as the boundary for growing endothelium and podocytes. Media flow is supplied through a supplying machine and connected to a vacuum chamber to induce cell stretching, shear stress, and compressing. Implementing this machine requires specialized accessory machinery, which complicates the setup [13]. The second model, like the one we studied, employs a bilateral flow with a central gel channel device. However, cells were prepared through primary culture; the cell maturation period took up to 15 or 28 days [1,14]. Lastly, the hollow alginate fiber devices do not require exogenous coating of the extracellular cellular matrix but continuously need CaCl_2_. A drawback of this method is that the cell maturation period can extend to 14 days [15]. The GFB-mimicking chip developed by our team features a simplified structure compared to conventional glomerulus-on-a-chip models, enhancing user-friendliness and ease of fabrication. This design enables the easy culture of the immortalized podocytes cell line and facilitates the induction of differentiation within a chip. Additionally, the 3D-MOTIVE chip we employed is designed to enable the simultaneous execution of three repetitive cultures on a single plate. It is sufficient for evaluating not only cell viability but also albumin permeability as a functional marker, like previous research on glomerulus-on-a-chip systems. Our approach achieved podocyte differentiation in just 8 days. This highlights the significant strengths of our model, enabling the assessment of disease models using puromycin and serum and showcasing a robust and versatile platform.

Podocytes in GFB are exposed to an environment where they experience shear stress due to the continuous flow of urine [16]. To simulate this phenomenon in vitro, we created a flow and confirmed in a previous study that the fluid shear stress calculated was like physiologic conditions [7]. However, in the human kidney, urinary or blood flow is not bidirectional. The bidirectional pulsatile flow used in our model by rockers did not replicate the unilateral fluid flow found in the kidney. Technically, to create a unidirectional flow, we could consider using an artificial pump, but this might lead to the formation of bubbles in the microfluidic channel, cell sedimentation, or cell lysis [17,18,19]. Therefore, despite the limitation of using a bidirectional flow, we opted for a gravity-driven flow approach to reduce the complexity of the device and cellular damage. In a preclinical setting, yet aiming for a closer simulation of the clinical environment, we attempted co-culture with endothelial cells and maintained a microfluidic environment in the GFB-mimicking chip. We compared the differentiation of podocytes under 2D culture conditions, and the results revealed not only an increase in WT1 expression concurrent with differentiation but also more pronounced differentiation morphology in the GFB-mimicking chip [8,11]. Vascular endothelial growth factor (VEGF) plays a crucial role in regulating the structure and function of glomerular endothelial cells to maintain the integrity of the GFB [20]. Paracrine signaling between podocytes and endothelial cells through VEGF-VEGFR-2 is essential for the development and maintenance of the GFB. This implies that co-culture and fluidic conditions in the GFB-mimicking chip can enhance the physiological relevance of the model, allowing for a more accurate representation of cellular interactions and functions as they occur in a clinical setting.

We used the GFB-mimicking chip to compare trends in a serum-induced MN model with clinical data from patients. An electron microscopy examination of kidney pathology revealed an inverse relationship between subepithelial cell EDD and cell viability decreases. Additionally, changes in uPCR post-tacrolimus treatment in patients were correlated with the reductions in albumin permeability induced by tacrolimus on the GFB-mimicking chip. However, full replication of uPCR occurred only in MN1 and MN5 and was attributed to the pronounced correlation observed in EDD changes, specifically in MN1 and MN2. The correlation between therapeutic effects in clinical data, pathological findings, and the serum-induced MN model showcases this model’s potential as a future preclinical screening tool for therapeutic effects of tacrolimus.

WT1 is known to be an important marker for podocyte maturation, functioning as a nuclear transcriptional factor by binding to the promoter region of nephrin cDNA to increase nephrin expression [21]. Nephrin plays a crucial role in maintaining the normal structure of the slit diaphragm in podocytes [11]. The expression of both WT1 and nephrin appears to be critical for preserving the structure and function of podocytes. Indeed, reports indicate a decrease in the expression of WT1 and nephrin in patients with minimal change disease (MCD) and focal segmental glomerulosclerosis (FSGS), suggesting a correlation between the expression of WT1 and nephrin in podocyte function in nephrotic syndrome [22]. In the context of this research, PAN was found to decrease the expression of both WT1 and nephrin, whereas treatment with tacrolimus increased WT1 expression but had a minimal impact on nephrin expression. In the MN1 serum-induced model, WT1, which decreased after serum treatment, increased with tacrolimus, whereas the expression of nephrin was not significantly different before and after tacrolimus treatment. The results may be indicative of a delayed restorative response of nephrin due to the impact on podocytes following glomerulonephritis induction. A study of glomerulogenesis from embryo to postnatal day revealed that WT1 was expressed first, followed by nephrin expression [23]. Consequently, it is speculated that nephrin recovery may be possible in the late stage of podocyte restoration. As 3D glomeruli tissue chips have limitations in observing long-term cellular changes, future research should focus on confirming the recovery of nephrin and investigating how its changes correlate with the restoration of normal GFB function.

As previously introduced in other literature, MN induction was achieved using serum from MN patients [1]. We have implemented the MN disease model in a similar manner, where humoral factors present in the disease patient serum were responsible for inducing MN. It is known that in the actual progression of MN, the activation of lymphocytes and the resulting positive feedback play an important role in the development of the disease, following the initial induction by humoral factors [24]. Our study’s limitations include excluding the role of lymphocytes in the pathophysiology of MN. While immunosuppressive agents targeting lymphocyte activation have traditionally been used in glomerular diseases, recent theories propose their direct and lymphocyte-independent reverse effects in restoring podocytopathy. Tacrolimus minimizes renal tissue damage by inhibiting the expression of transient receptor potential cation channel-6 (TRPC6) and impeding T cell activation. Podocytes cultured independently in a 2D environment and subjected to puromycin-induced injury showed enhanced autophagy after tacrolimus treatment by increasing the expression of microtubule-associated proteins 1A/1B light chain 3A (LC3), thereby preventing renal damage [25]. In rodent models of PAN-induced podocyte injury, the administration of tacrolimus confirmed the restoration of podocyte foot processes. It also demonstrated protection against PAN-induced injury in vitro through the pretreatment of podocytes with tacrolimus. This protection is attributed to the inhibition of the mitogen-activated protein kinase (MAPK) pathway and an anti-apoptotic mechanism [26].

This platform, leveraging MN models with the GFB-mimicking chip, significantly advances personalized treatment strategies by providing the capability to predict the efficacy of tacrolimus for specific MN patients. By assessing the potential for tacrolimus to improve proteinuria, this approach not only tailors therapy to the unique disease characteristics of each patient but also streamlines the process of treatment selection, thereby reducing the time needed to identify the most effective treatment option. As a result, the reduction in proteinuria due to the selection of appropriate treatment ultimately improves the patient’s prognosis. Nevertheless, it will be necessary to replicate the efficacy of not only tacrolimus but also other therapeutic agents using the MN model in the GFB-mimicking chip in the future. The podocyte cell line CIHP-1 that we used is reported to be culturable for up to 2 weeks [27]. However, our 3D-MOTIVE chip faced a limitation in cell culture space, leading to cell overcrowding and posing challenges for extended culture periods. Therefore, we are considering enlarging the plate size as a potential solution. This adjustment would enable the study of the long-term effects on the glomerular filtration barrier (GFB). Additionally, we are currently investigating other therapeutic agents using the MN model and developing various disease models on the standard GFB-mimicking chip. This approach seems promising to enhance its reliability in the future through comparative analysis with clinical data. Our experimental model will offer a physiological perspective for studying molecular changes in podocytes and morphological changes, such as slip diaphragm alterations. Although we did not observe variations in the MN model within the GFB-mimicking chip based on the presence or absence of anti-PLA2R antibodies in patient serum, the absence of anti-PLA2R antibodies in serum does not necessarily rule out their involvement as a causative factor [28]. There have been instances where PLA2R was confirmed in glomerular deposits through actual tissue examination, even when antibodies were not detected in serum. Conversely, there were cases of serum positivity without corresponding glomerular deposits.

Thus, it is essential to validate the correlation with clinical outcomes to further develop the GFB-mimicking chip as a preclinical platform for drug efficacy testing. We assessed the relevance of the severity of pathological changes in five samples on the chip. However, a more extensive comparison with a larger sample size is warranted for comprehensive validation.

## 5. Conclusions

We successfully evaluated the efficacy of tacrolimus using puromycin-induced and serum-induced GN models on a chip that mimics the structure and function of the GFB. The GFB-mimicking chip holds promise as a personalized platform for assessing drug efficacy using patient serum samples.

## Figures and Tables

**Table 1:** General characteristics and outcomes of patients with membranous nephropathy.

	MN1	MN2	MN3	MN4	MN5
Sex	female	female	male	female	male
SBP (mmHg)	128	129	120	145	147
DBP (mmHg)	77	66	62	75	73
WBC (10^3^/μL)	10,300	7700	7200	4810	6230
Hemoglobin (g/dL)	12.9	12	12	13	10.6
Platelets (10^3^/μL)	237	391	339	298	211
Peak UPCR (mg/g)	4551	5373	10,859.14	9225.91	21,393.15
Serum creatinine (mg/dL)	0.53	0.62	0.83	1.09	1.59
eGFR-MDRD (mL/min/1.73 m^2^)	120	105.8	95.8	122.2	47.2
Anti-PLA2R antibody (RU/mL)	neg	41.2	55.6	146.7	neg
Treatment					
RAS blockade	+	+	+	+	+
Steroid + tacrolimus	+	+	+	−	+
Pathology					
H&E					
Capillary wall thickening	diffuse	diffuse	diffuse	diffuse	diffuse
IF					
Glomerulus IgG deposits	2+	2+	2+	2+	3+
EM					
GBM thickening	diffuse	diffuse	diffuse	diffuse	diffuse
PFE	diffuse	diffuse	diffuse	diffuse	diffuse
EDD, subepithelial	large	large	moderate	moderate	moderate
Outcome after tacrolimus					
UPCR (mg/g)	164.15	891.76	10,859.14	.	495.84
Serum creatinine (mg/dL)	0.59	1.2	1.41	.	1.12

Abbreviations: SBP = systolic blood pressure; DBP = diastolic blood pressure; UPCR = urine–protein–creatinine ratio; PFE = podocyte foot process effacement; EDD = electrodense deposit. ‘+’ indicates a history of drug use, while ‘−’ indicates no history of drug use.

## Data Availability

The data presented in this study are available on request from the corresponding author. The data are not publicly available due to privacy.

## References

[1] Petrosyan A., Cravedi P., Villani V., Angeletti A., Manrique J., Renieri A., De Filippo R.E., Perin L., Da Sacco S. (2019). A glomerulus-on-a-chip to recapitulate the human glomerular filtration barrier. Nat. Commun..

[2] Rojas-Rivera J., Fervenza F.C., Ortiz A. (2022). Recent Clinical Trials Insights into the Treatment of Primary Membranous Nephropathy. Drugs.

[3] Van Norman G.A. (2019). Limitations of Animal Studies for Predicting Toxicity in Clinical Trials: Is it Time to Rethink Our Current Approach?. JACC Basic Transl. Sci..

[4] Kwon E.J., Hwang S.H., Seo S., Park J., Park S., Kim S. (2023). Efficacy of Mesenchymal-Stromal-Cell-Derived Extracellular Vesicles in Ameliorating Cisplatin Nephrotoxicity, as Modeled Using Three-Dimensional, Gravity-Driven, Two-Layer Tubule-on-a-Chip (3D-MOTIVE Chip). Int. J. Mol. Sci..

[5] Kim K., Jeong B., Lee Y.-M., Son H.-E., Ryu J.-Y., Park S., Jeong J.C., Chin H.J., Kim S. (2022). Three-Dimensional Kidney-on-a-Chip Assessment of Contrast-Induced Kidney Injury: Osmolality and Viscosity. Micromachines.

[6] Vormann M.K., Gijzen L., Hutter S., Boot L., Nicolas A., van den Heuvel A., Vriend J., Ng C.P., Nieskens T.T.G., van Duinen V. (2018). Nephrotoxicity and Kidney Transport Assessment on 3D Perfused Proximal Tubules. AAPS J..

[7] Ogonczyk D., Wegrzyn J., Jankowski P., Dabrowski B., Garstecki P. (2010). Bonding of microfluidic devices fabricated in polycarbonate. Lab Chip.

[8] Shankland S.J., Pippin J.W., Reiser J., Mundel P. (2007). Podocytes in culture: Past, present, and future. Kidney Int..

[9] Dong N., Meng L., Xue R., Yu M., Zhao Z., Liu X. (2017). Adrenomedullin ameliorates podocyte injury induced by puromycin aminonucleoside in vitro and in vivo through modulation of Rho GTPases. Int. Urol. Nephrol..

[10] Jiang L., Cui H., Ding J., Yang A., Zhang Y. (2020). Puromycin aminonucleoside-induced podocyte injury is ameliorated by the Smad3 inhibitor SIS3. FEBS Open Bio.

[11] Krtil J., Pláteník J., Kazderová M., Tesar V., Zima T. (2007). Culture methods of glomerular podocytes. Kidney Blood Press. Res..

[12] Doi K., Kimura H., Matsunaga Y.T., Fujii T., Nangaku M. (2022). Glomerulus-on-a-Chip: Current Insights and Future Potential Towards Recapitulating Selectively Permeable Filtration Systems. Int. J. Nephrol. Renovasc Dis..

[13] Roye Y., Bhattacharya R., Mou X., Zhou Y., Burt M.A., Musah S. (2021). A Personalized Glomerulus Chip Engineered from Stem Cell-Derived Epithelium and Vascular Endothelium. Micromachines.

[14] Musah S., Mammoto A., Ferrante T.C., Jeanty S.S.F., Hirano-Kobayashi M., Mammoto T., Roberts K., Chung S., Novak R., Ingram M. (2017). Mature induced-pluripotent-stem-cell-derived human podocytes reconstitute kidney glomerular-capillary-wall function on a chip. Nat. Biomed. Eng..

[15] Xie R., Korolj A., Liu C., Song X., Lu R.X.Z., Zhang B., Ramachandran A., Liang Q., Radisic M. (2020). h-FIBER: Microfluidic Topographical Hollow Fiber for Studies of Glomerular Filtration Barrier. ACS Cent. Sci..

[16] Kriz W., Lemley K.V. (2017). Potential relevance of shear stress for slit diaphragm and podocyte function. Kidney Int..

[17] Huh D., Matthews B.D., Mammoto A., Montoya-Zavala M., Hsin H.Y., Ingber D.E. (2010). Reconstituting organ-level lung functions on a chip. Science.

[18] Moura Rosa P., Gopalakrishnan N., Ibrahim H., Haug M., Halaas Ø. (2016). The intercell dynamics of T cells and dendritic cells in a lymph node-on-a-chip flow device. Lab Chip.

[19] Van Os L., Engelhardt B., Guenat O.T. (2023). Integration of immune cells in organs-on-chips: A tutorial. Front. Bioeng. Biotechnol..

[20] Sison K., Eremina V., Baelde H., Min W., Hirashima M., Fantus I.G., Quaggin S.E. (2010). Glomerular structure and function require paracrine, not autocrine, VEGF-VEGFR-2 signaling. J. Am. Soc. Nephrol..

[21] Ettou S., Jung Y.L., Miyoshi T., Jain D., Hiratsuka K., Schumacher V., Taglienti M.E., Morizane R., Park P.J., Kreidberg J.A. (2020). Epigenetic transcriptional reprogramming by WT1 mediates a repair response during podocyte injury. Sci. Adv..

[22] Pereira L.H.M., da Silva C.A., Monteiro M., Araújo L.S., Rocha L.P., Reis M., Ramalho F.S., Corrêa R.R.M., Silva M.V., Reis M.A. (2019). Podocin and uPAR are good biomarkers in cases of Focal and segmental glomerulosclerosis in pediatric renal biopsies. PLoS ONE.

[23] Kato T., Mizuno S. (2017). Nephron, Wilms’ tumor-1 (WT1), and synaptopodin expression in developing podocytes of mice. Exp. Anim..

[24] Lin D.-W., Chang C.-C., Hsu Y.-C., Lin C.-L. (2022). New Insights into the Treatment of Glomerular Diseases: When Mechanisms Become Vivid. Int. J. Mol. Sci..

[25] Yang X.Q., Yu S.Y., Yu L., Ge L., Zhang Y., Hao Z.H., Liu G.S. (2020). Effects of tacrolimus on autophagy protein LC3 in puromycin-damaged mouse podocytes. J. Int. Med. Res..

[26] Shen X., Jiang H., Ying M., Xie Z., Li X., Wang H., Zhao J., Lin C., Wang Y., Feng S. (2016). Calcineurin inhibitors cyclosporin A and tacrolimus protect against podocyte injury induced by puromycin aminonucleoside in rodent models. Sci. Rep..

[27] Da Sacco S., Lemley K.V., Sedrakyan S., Zanusso I., Petrosyan A., Peti-Peterdi J., Burford J., De Filippo R.E., Perin L. (2013). A novel source of cultured podocytes. PLoS ONE.

[28] Debiec H., Ronco P. (2011). PLA2R autoantibodies and PLA2R glomerular deposits in membranous nephropathy. N. Engl. J. Med..

